# Supplementary material for: The impact of package selection and versioning on single-cell RNA-seq analysis
Source: bioRxiv. 2024 Apr 11:2024.04.04.588111. Preprint. [Version 2] doi: 10.1101/2024.04.04.588111 (PMC11014608; doi:10.1101/2024.04.04.588111)
Supplement: Supplement 1 [file media-1.pdf]

Supplemental Information
The impact of package selection and versioning on single-cell RNA-seq analysis

Joseph M Rich[1,2], Lambda Moses[1], Pétur Helgi Einarsson[3], Kayla Jackson[1,2], Laura Luebbert[1], A. Sina Booeshaghi[1], Sindri Antonsson[3], Delaney K. Sullivan[1,4], Nicolas Bray[5], Páll Melsted[3], and Lior Pachter*[1,6,7]

[1]*Biology and Biological Engineering, California Institute of Technology, Pasadena, CA, 91125, USA*

[2]*USC-Caltech MD/PhD Program, Keck School of Medicine, Los Angeles, CA, 90033, USA*

[3]*Faculty of Industrial Engineering, Mechanical Engineering and Computer Science, Reykjavík, Iceland*

[4]*UCLA-Caltech Medical Scientist Training Program, David Geffen School of Medicine, University of California, Los Angeles, Los Angeles, CA, 90095, USA*

[5]*Boston, MA*

[6]*Computing and Mathematical Sciences, California Institute of Technology, Pasadena, CA, 91125, USA*

[7]*Lead Contact*

April 9, 2024

*Correspondence: lpachter@caltech.edu.

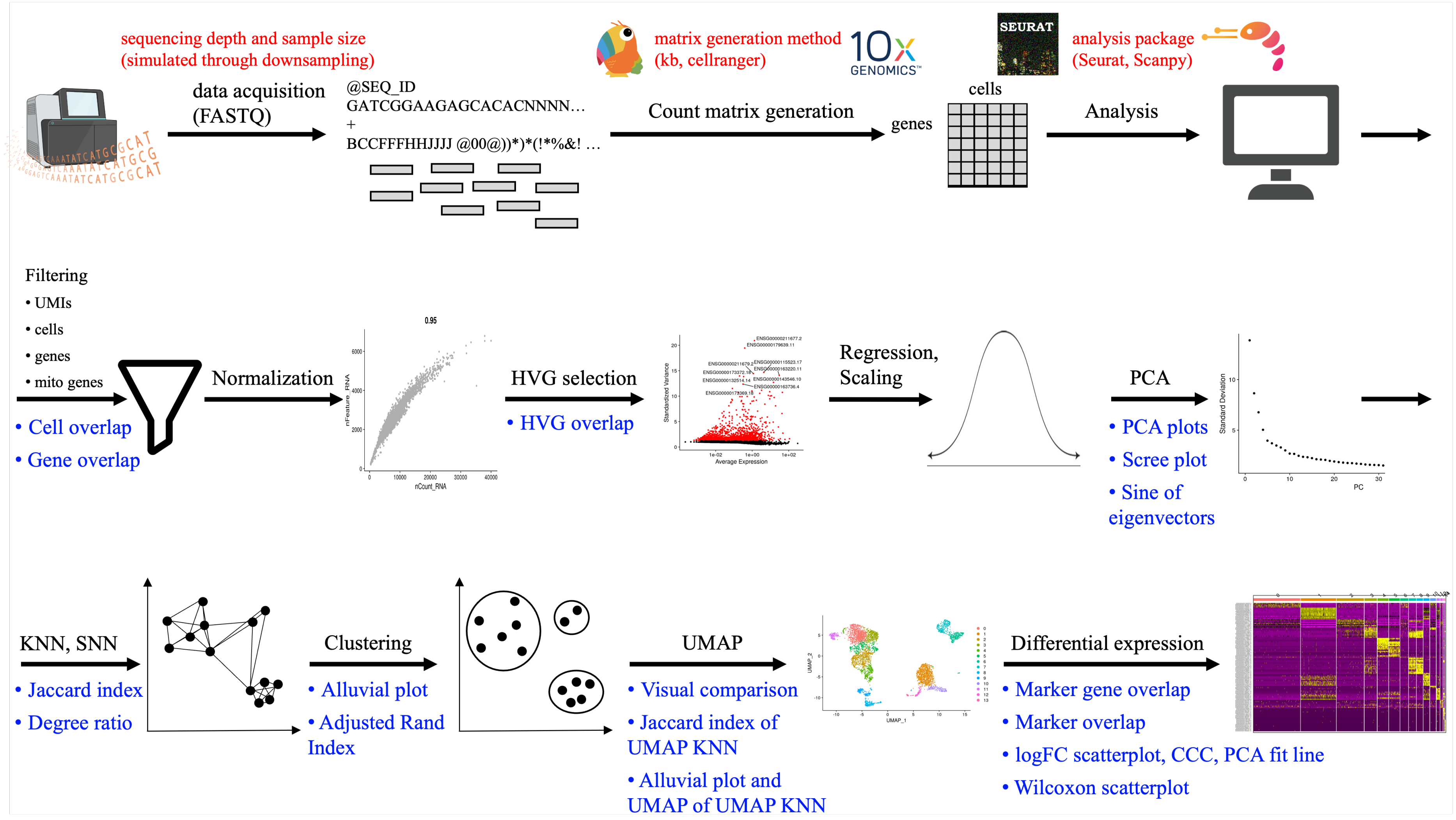

sequencing depth and sample size
(simulated through downsampling)
data acquisition
(FASTQ)
@SEQ_ID
GATCGGAAGAGCACACNNNN…
+
BCCFFFHHJJJJ @00@))*)*(!*%&! …
matrix generation method
(kb, cellranger)
10x GENOMICS
Count matrix generation
genes
cells
SEURAT
analysis package
(Seurat, Scanpy)
Analysis
Filtering
• UMIs
• cells
• genes
• mito genes
• Cell overlap
• Gene overlap
Normalization
HVG selection
• HVG overlap
Regression,
Scaling
PCA
• PCA plots
• Scree plot
• Sine of eigenvectors
Standard Deviation
PC
KNN, SNN
• Jaccard index
• Degree ratio
Clustering
• Alluvial plot
• Adjusted Rand Index
UMAP
• Visual comparison
• Jaccard index of UMAP KNN
• Alluvial plot and UMAP of UMAP KNN
Differential expression
• Marker gene overlap
• Marker overlap
• logFC scatterplot, CCC, PCA fit line
• Wilcoxon scatterplot

Supplemental Figure 1: scRNA-seq matrix generation and analysis overview. Red = sources of variability explored in this study; Blue = plots and metrics used to assess function similarity

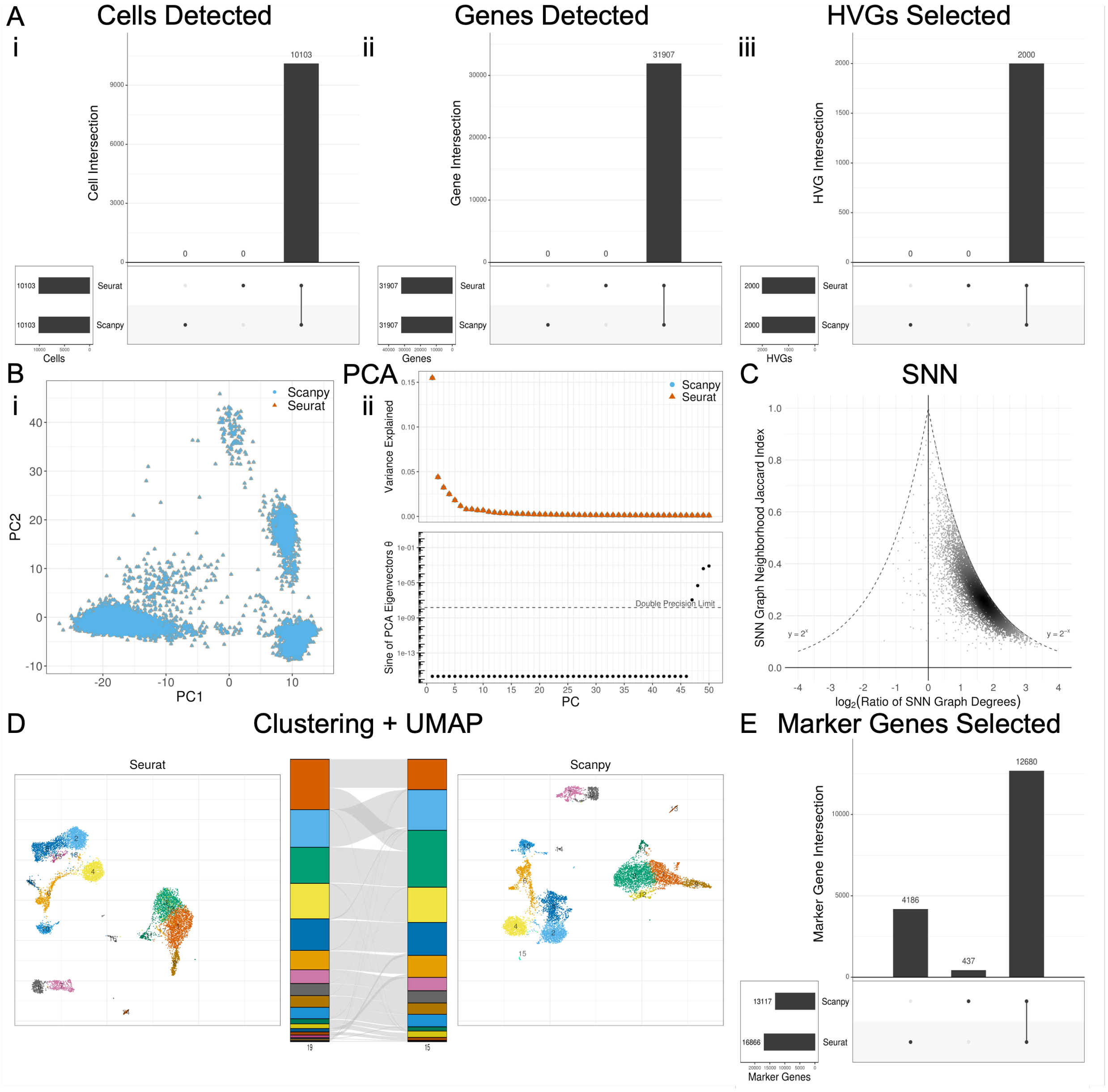

Supplemental Figure 2: Seurat vs. Scanpy, aligned function arguments (Seurat-like), sequential analysis. (A) Filtering and HVG selection analysis UpSet plots consisting of overlap of sets of cells, genes, and HVGs. (B) PCA analysis through projection onto first 2 PCs, Scree plot eigenvalue comparison, and sine of eigenvectors. Black lines = point mapping between conditions. (C) KNN/SNN analysis through SNN neighborhood Jaccard index and degree ratio (Seurat/Scanpy) per cell. (D) Clustering and UMAP analysis through UMAP plots of each condition, with alluvial plot showing cluster assignment mapping and degree of agreement. (E) Differential expression analysis UpSet plot through overlap of all significant ($p < 0.05$) marker genes across all clusters.

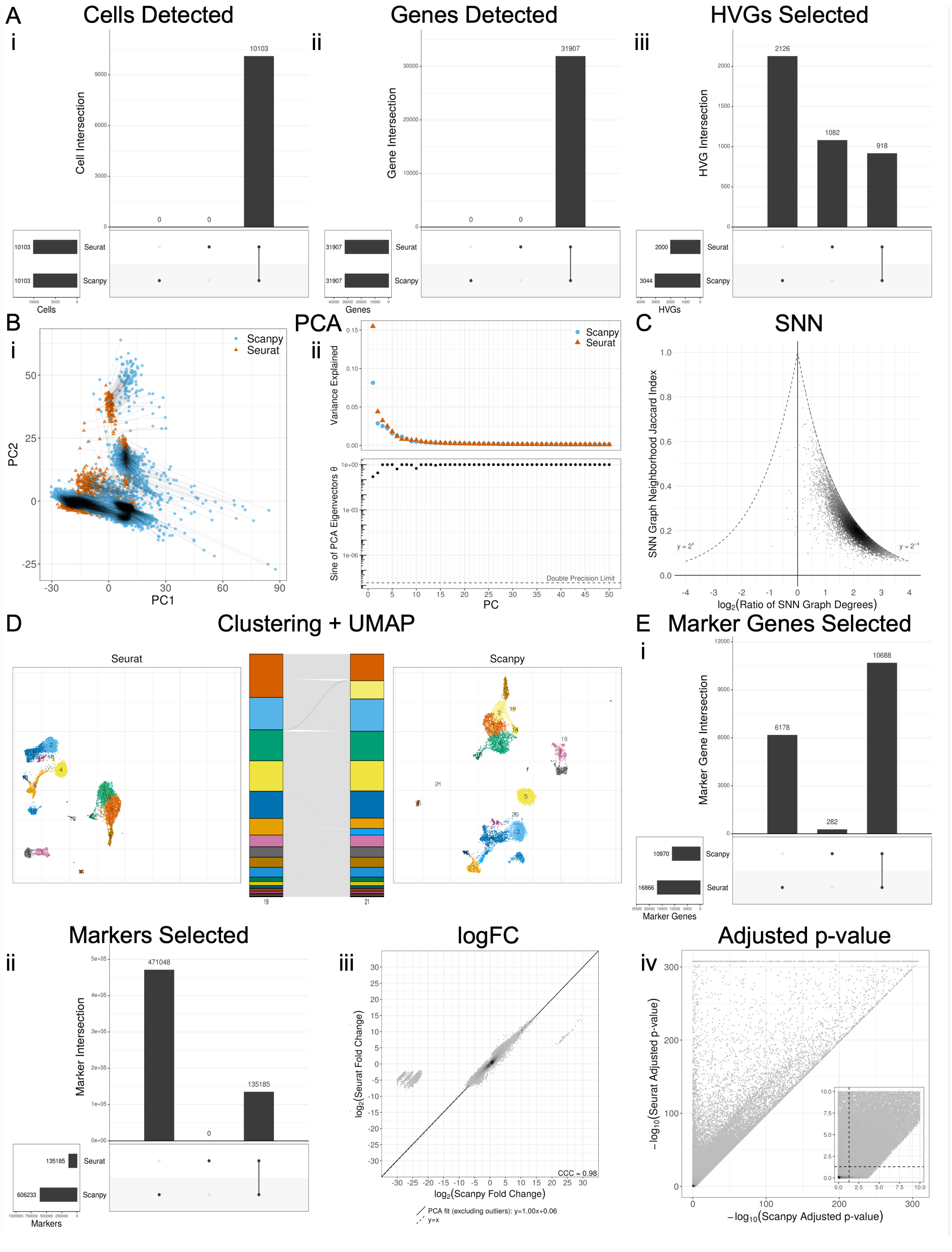
A
Cells Detected
Genes Detected
HVGs Selected
i
ii
iii
Cell Intersection
Gene Intersection
HVG Intersection
10103
0
0
31907
0
0
2126
1082
918
Seurat
Scanpy
Cells
Genes
HVGs
B
PCA
C
SNN
Scanpy
Seurat
PC1
PC2
Variance Explained
Sine of PCA Eigenvectors θ
Double Precision Limit
PC
SNN Graph Neighborhood Jaccard Index
log₂(Ratio of SNN Graph Degrees)
D
Clustering + UMAP
Seurat
Scanpy
E
Marker Genes Selected
Marker Gene Intersection
6178
282
10688
10970
16866
Marker Genes
Markers Selected
Marker Intersection
471048
0
135185
606233
Markers
logFC
iv
Adjusted p-value
log₂(Seurat Fold Change)
log₂(Scanpy Fold Change)
CCC = 0.98
PCA fit (excluding outliers): y=1.00x+0.06
y=x
−log₁₀(Seurat Adjusted p-value)
−log₁₀(Scanpy Adjusted p-value)

Supplemental Figure 3: Seurat vs. Scanpy, default function arguments, controlled analysis. (A) Filtering and HVG selection analysis UpSet plots consisting of overlap of sets of cells, genes, and HVGs. (B) PCA analysis through projection onto first 2 PCs, Scree plot eigenvalue comparison, and sine of eigenvectors. Black lines = point mapping between conditions. (C) KNN/SNN analysis through SNN neighborhood Jaccard index and degree ratio (Seurat/Scanpy) per cell. (D) Clustering and UMAP analysis through UMAP plots of each condition, with alluvial plot showing cluster assignment mapping and degree of agreement. (E) Differential expression analysis through overlap of all significant ($p < 0.05$) marker genes across all clusters. (F) Differential expression analysis with aligned clusters of overlap of all markers. (G) Differential expression analysis with aligned clusters of logFC similarity. (H) Differential expression analysis with aligned clusters of adjusted p-value similarity.

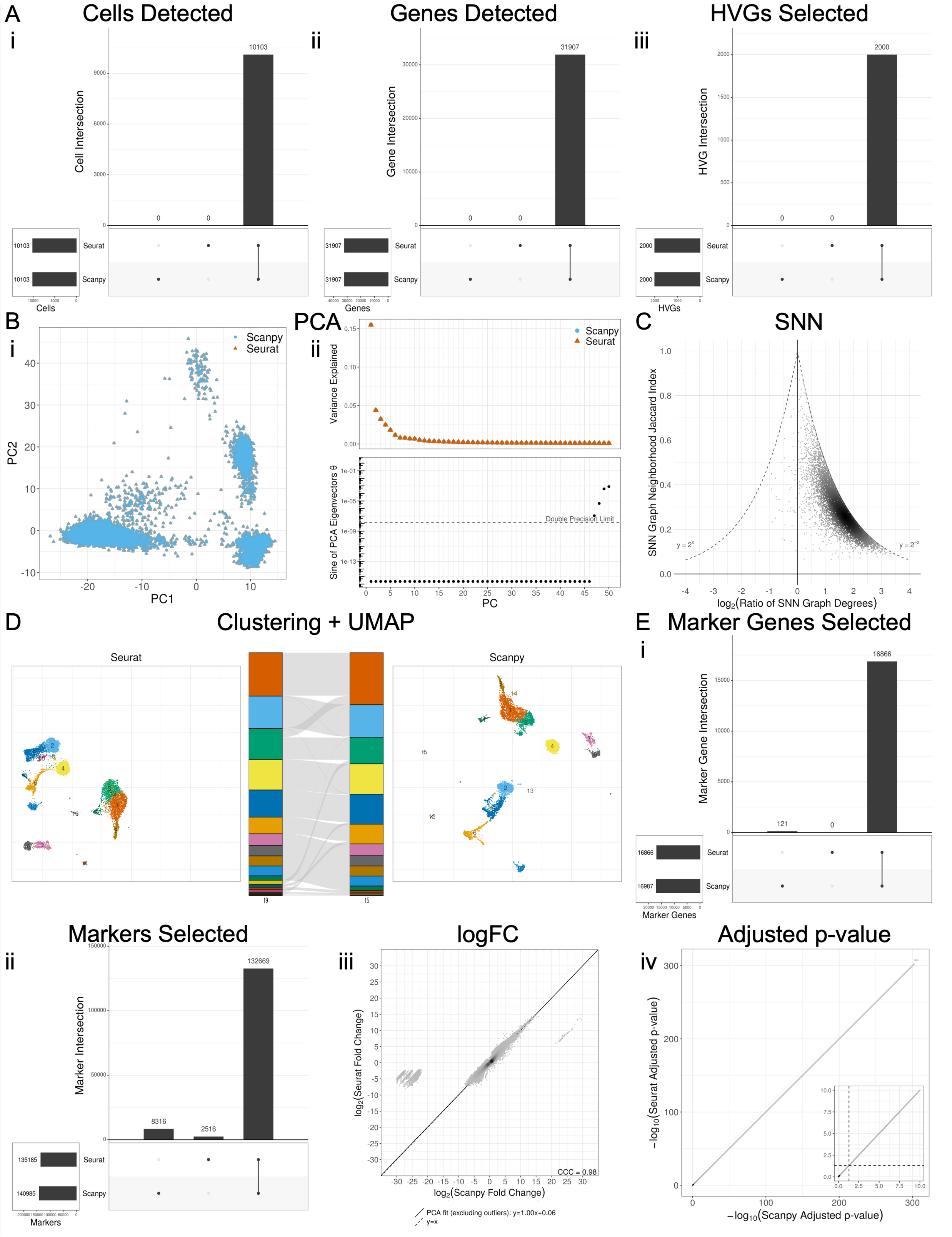
A
Cells Detected
i
Cell Intersection
Cells
Seurat
Scanpy
Genes Detected
ii
Gene Intersection
Genes
HVGs Selected
iii
HVG Intersection
HVGs
B
i
PC1
PC2
PCA
ii
Variance Explained
Sine of PCA Eigenvectors θ
Double Precision Limit
PC
C
SNN
SNN Graph Neighborhood Jaccard Index
log2(Ratio of SNN Graph Degrees)
D
Clustering + UMAP
E
Marker Genes Selected
Marker Gene Intersection
Marker Genes
Markers Selected
Marker Intersection
Markers
logFC
log2(Seurat Fold Change)
log2(Scanpy Fold Change)
CCC = 0.98
PCA fit (excluding outliers): y=1.00x+0.06
y=x
Adjusted p-value
iv
−log10(Seurat Adjusted p-value)
−log10(Scanpy Adjusted p-value)

Supplemental Figure 4: Seurat vs. Scanpy, aligned function arguments (Seurat-like), controlled analysis. (A) Filtering and HVG selection analysis UpSet plots consisting of overlap of sets of cells, genes, and HVGs. (B) PCA analysis through projection onto first 2 PCs, Scree plot eigenvalue comparison, and sine of eigenvectors. Black lines = point mapping between conditions. (C) KNN/SNN analysis through SNN neighborhood Jaccard index and degree ratio (Seurat/Scanpy) per cell. (D) Clustering and UMAP analysis through UMAP plots of each condition, with alluvial plot showing cluster assignment mapping and degree of agreement. (E) Differential expression analysis through overlap of all significant ($p < 0.05$) marker genes across all clusters. (F) Differential expression analysis with aligned clusters of overlap of all markers. (G) Differential expression analysis with aligned clusters of logFC similarity. (H) Differential expression analysis with aligned clusters of adjusted p-value similarity.

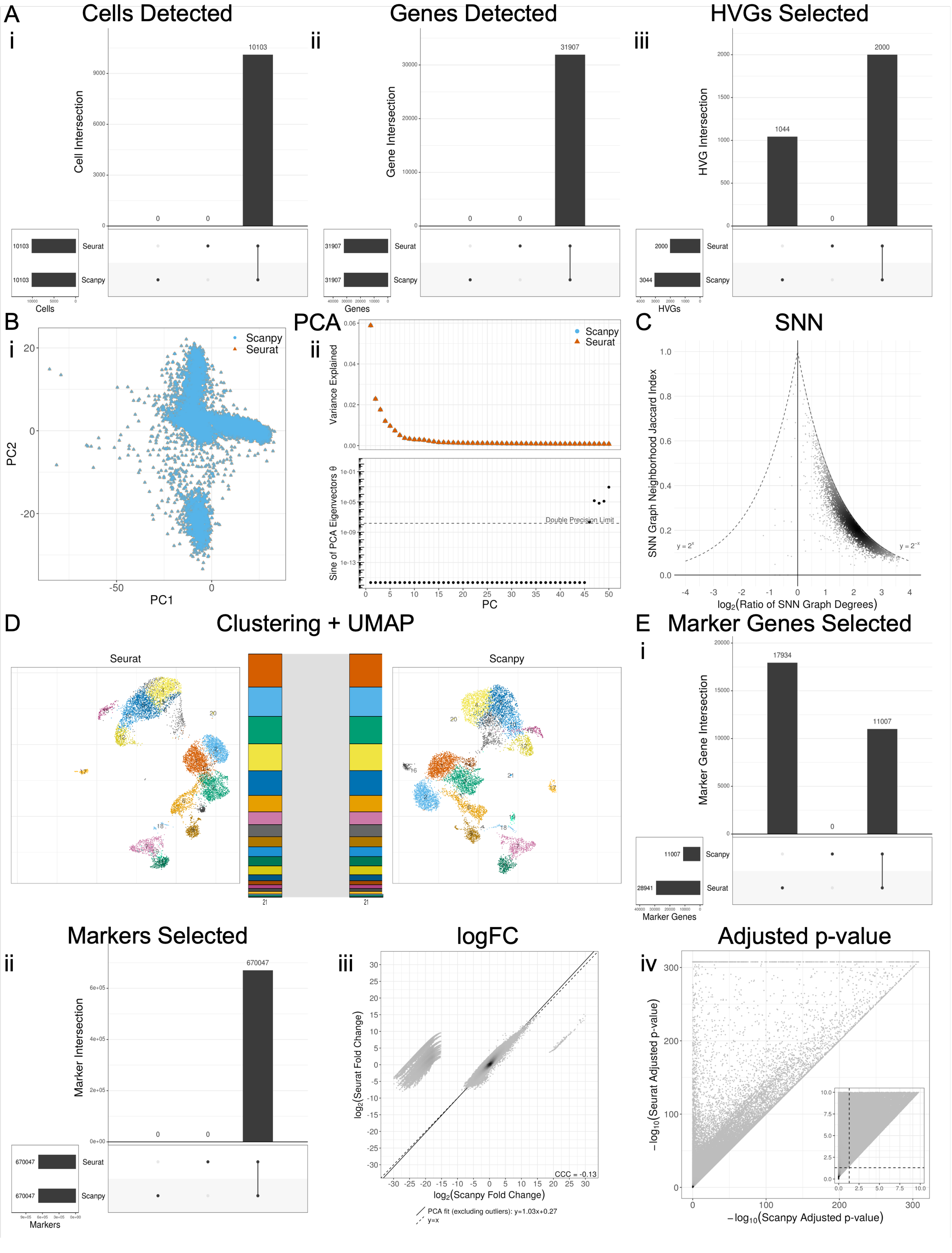

A
Cells Detected
i
Cell Intersection
Cells
Seurat
Scanpy
10103
Genes Detected
ii
Gene Intersection
Genes
31907
HVGs Selected
iii
HVG Intersection
HVGs
2000
1044
3044
B
i
Scanpy
Seurat
PC1
PC2
PCA
ii
Variance Explained
Sine of PCA Eigenvectors θ
Double Precision Limit
PC
C
SNN
SNN Graph Neighborhood Jaccard Index
log2(Ratio of SNN Graph Degrees)
D
Clustering + UMAP
Seurat
Scanpy
E
Marker Genes Selected
i
Marker Gene Intersection
Marker Genes
17934
11007
28941
Markers Selected
ii
Marker Intersection
Markers
670047
logFC
iii
log2(Seurat Fold Change)
log2(Scanpy Fold Change)
CCC = -0.13
PCA fit (excluding outliers): y=1.03x+0.27
y=x
Adjusted p-value
iv
−log10(Seurat Adjusted p-value)
−log10(Scanpy Adjusted p-value)

Supplemental Figure 5: Seurat vs. Scanpy, aligned function arguments (Scanpy-like), controlled analysis. (A) Filtering and HVG selection analysis UpSet plots consisting of overlap of sets of cells, genes, and HVGs. (B) PCA analysis through projection onto first 2 PCs, Scree plot eigenvalue comparison, and sine of eigenvectors. Black lines = point mapping between conditions. (C) KNN/SNN analysis through SNN neighborhood Jaccard index and degree ratio (Seurat/Scanpy) per cell. (D) Clustering and UMAP analysis through UMAP plots of each condition, with alluvial plot showing cluster assignment mapping and degree of agreement. (E) Differential expression analysis through overlap of all significant ($p < 0.05$) marker genes across all clusters. (F) Differential expression analysis with aligned clusters of overlap of all markers. (G) Differential expression analysis with aligned clusters of logFC similarity. (H) Differential expression analysis with aligned clusters of adjusted p-value similarity.

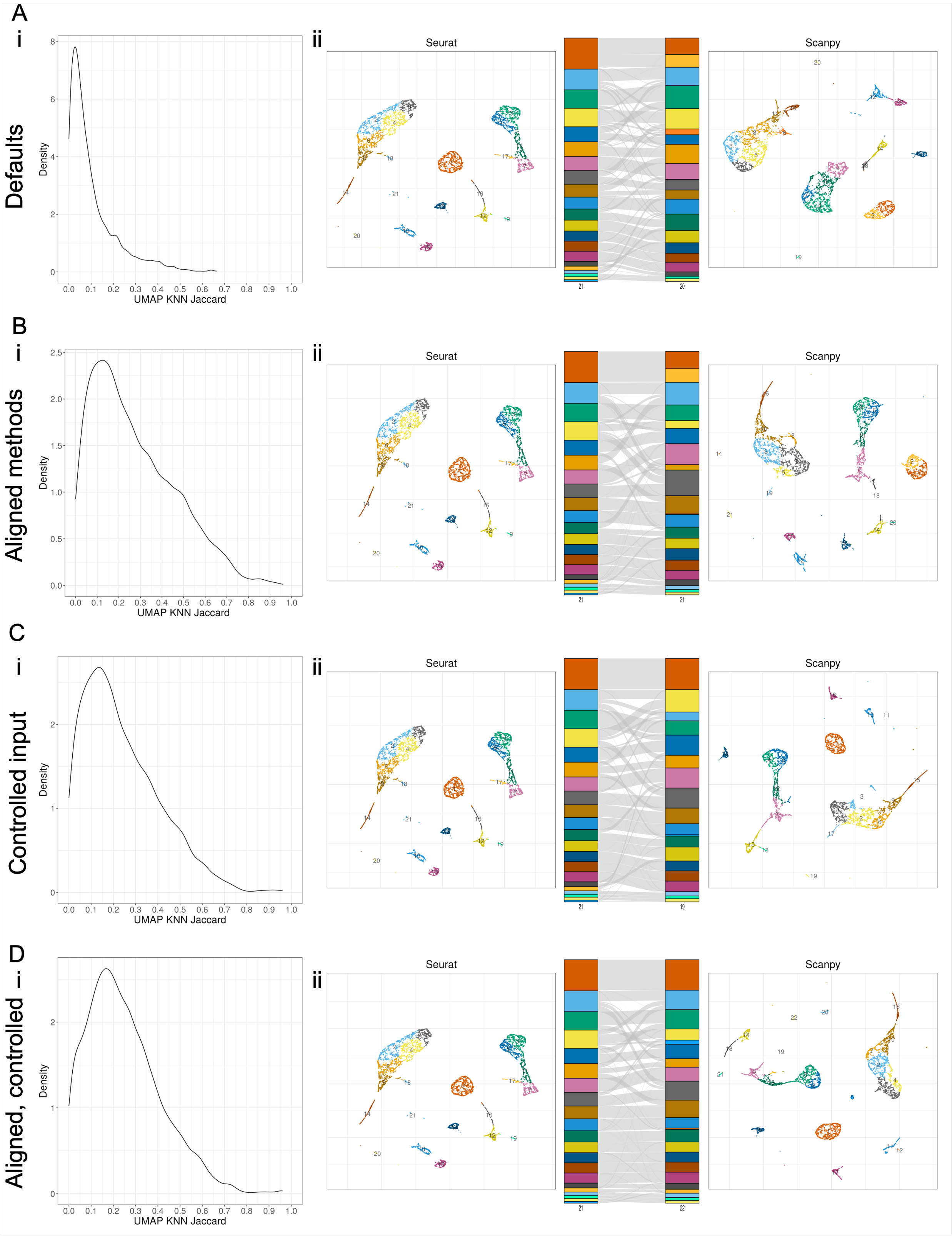

A
i
Defaults
ii
Seurat
Scanpy
B
i
Aligned methods
ii
Seurat
Scanpy
C
i
Controlled input
ii
Seurat
Scanpy
D
i
Aligned, controlled
ii
Seurat
Scanpy
Density
UMAP KNN Jaccard

Supplemental Figure 6: Extended UMAP analysis for Seurat vs. Scanpy showing density plot of UMAP-derived KNN graph neighborhood Jaccard indices, alluvial plot of leiden clustering performed on UMAP-derived KNN graph, and UMAP projection of UMAP-derived KNN graph colored with leiden clustering results. (A) Default methods, sequential analysis. (B) Aligned methods, sequential analysis. (C) Default methods, controlled analysis. (D) Aligned methods, controlled analysis.

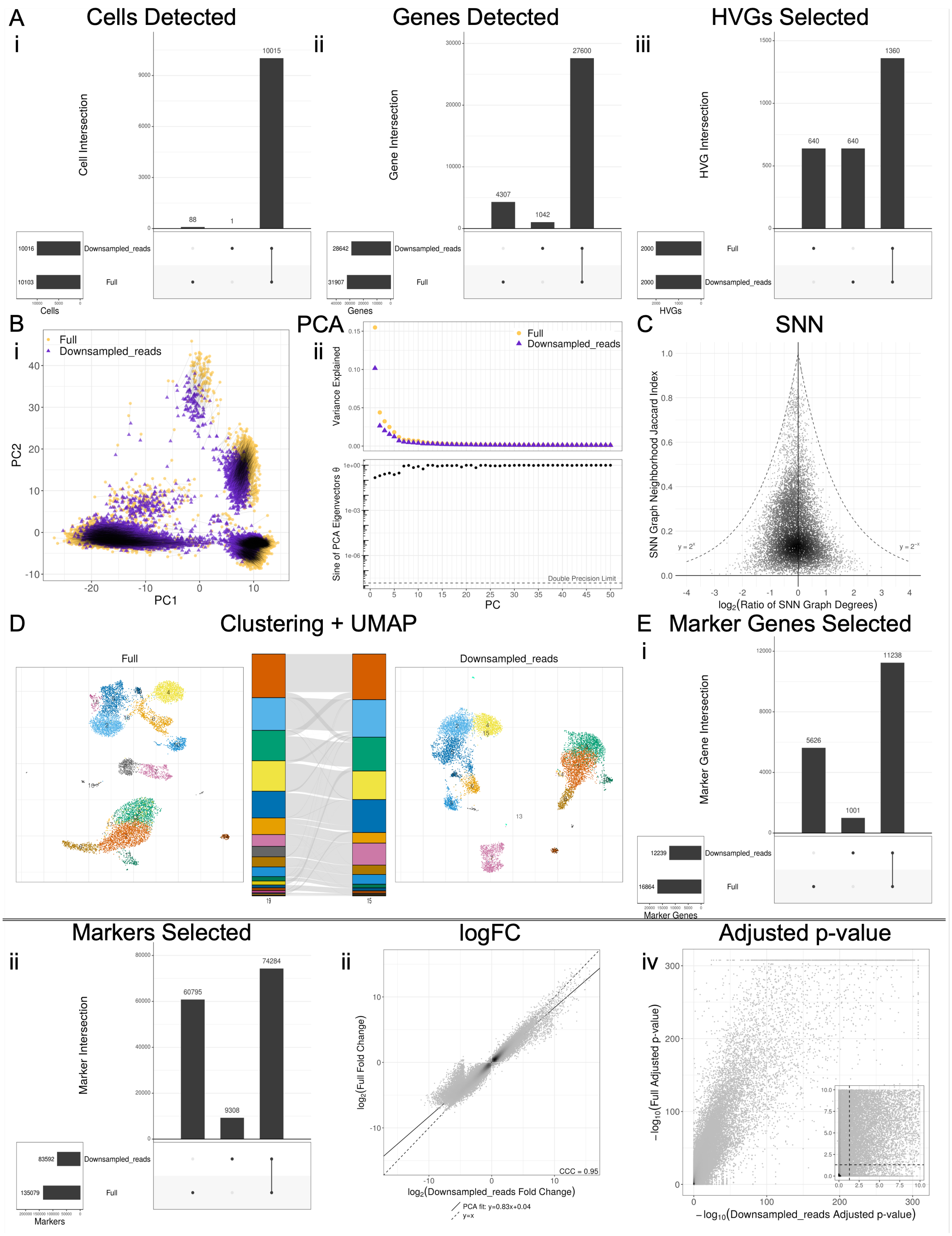

A
Cells Detected
i
Cell Intersection
88
1
10015
10016
Downsampled_reads
10103
Full
Cells
ii
Genes Detected
Gene Intersection
4307
1042
27600
28642
Downsampled_reads
31907
Full
Genes
iii
HVGs Selected
HVG Intersection
640
640
1360
2000
Full
2000
Downsampled_reads
HVGs
B
i
Full
Downsampled_reads
PC1
PC2
PCA
ii
Variance Explained
Sine of PCA Eigenvectors θ
Double Precision Limit
PC
C
SNN
SNN Graph Neighborhood Jaccard Index
log2(Ratio of SNN Graph Degrees)
D
Clustering + UMAP
Full
Downsampled_reads
E
Marker Genes Selected
i
Marker Gene Intersection
5626
1001
11238
12239
Downsampled_reads
16864
Full
Marker Genes
ii
Markers Selected
Marker Intersection
60795
9308
74284
83592
Downsampled_reads
135079
Full
Markers
logFC
ii
log2(Full Fold Change)
log2(Downsampled_reads Fold Change)
CCC = 0.95
PCA fit: y=0.83x+0.04
y=x
Adjusted p-value
iv
−log10(Full Adjusted p-value)
−log10(Downsampled_reads Adjusted p-value)

Supplemental Figure 7: Read downsampling to 4% of original size vs. full dataset in Seurat, with a fraction that displays variation comparable to Seurat vs. Scanpy. (A) Filtering and HVG selection analysis UpSet plots consisting of overlap of sets of cells, genes, and HVGs. (B) PCA analysis through projection onto first 2 PCs, Scree plot eigenvalue comparison, and sine of eigenvectors. Black lines = point mapping between conditions. (C) KNN/SNN analysis through SNN neighborhood Jaccard index and degree ratio (full/downsampled) per cell. (D) Clustering and UMAP analysis through UMAP plots of each condition, with alluvial plot showing cluster assignment mapping and degree of agreement. (E) Differential expression analysis through overlap of all significant ($p < 0.05$) marker genes across all clusters. (F) Differential expression analysis with aligned clusters of overlap of all markers. (G) Differential expression analysis with aligned clusters of logFC similarity. (H) Differential expression analysis with aligned clusters of adjusted p-value similarity.

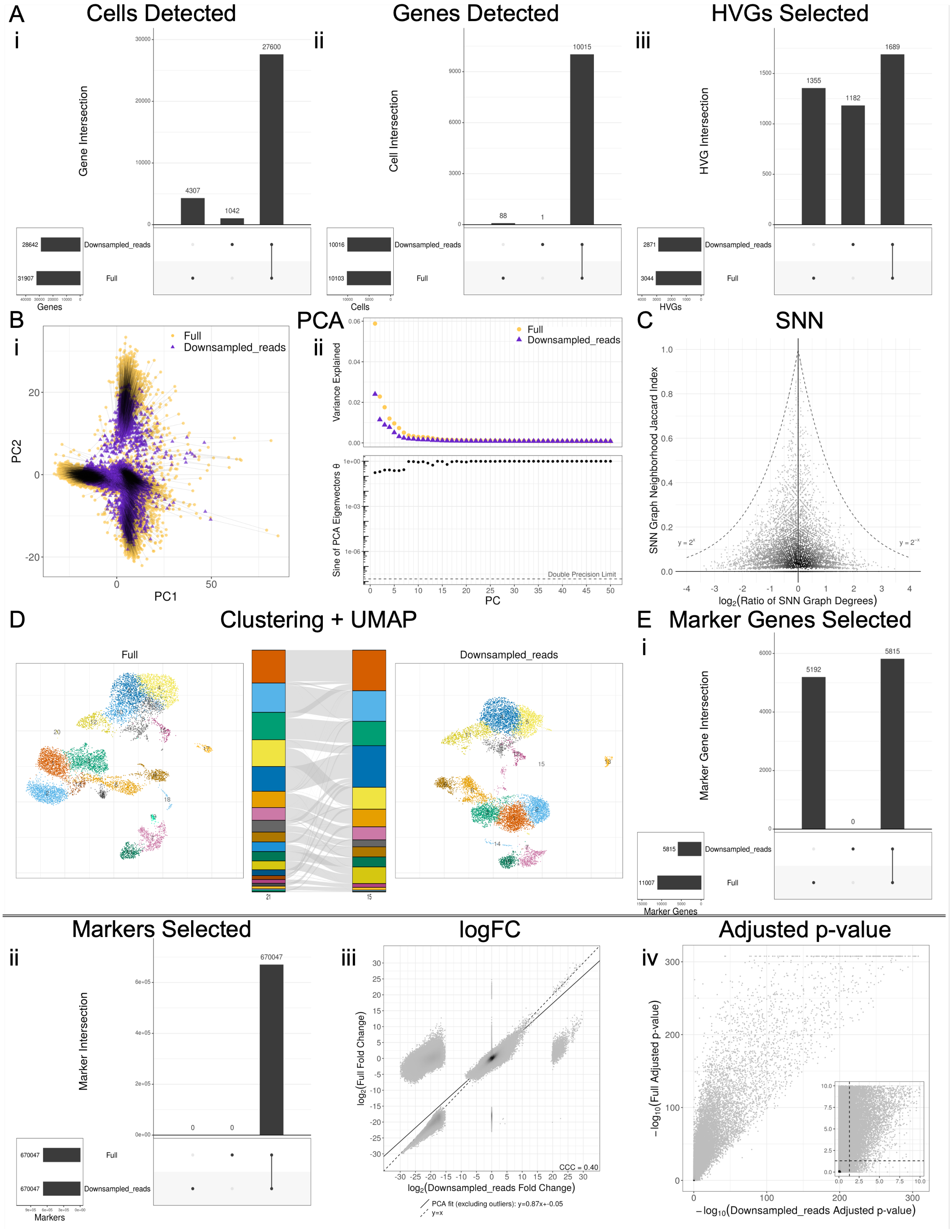

A
Cells Detected
i
Gene Intersection
4307
1042
27600
28642
Downsampled_reads
31907
Full
Genes
Genes Detected
ii
Cell Intersection
88
1
10015
10016
Downsampled_reads
10103
Full
Cells
HVGs Selected
iii
HVG Intersection
1355
1182
1689
2871
Downsampled_reads
3044
Full
HVGs
B
i
Full
Downsampled_reads
PC2
PC1
PCA
ii
Variance Explained
Full
Downsampled_reads
Sine of PCA Eigenvectors θ
Double Precision Limit
PC
C
SNN
SNN Graph Neighborhood Jaccard Index
y = 2^x
y = 2^-x
log2(Ratio of SNN Graph Degrees)
D
Clustering + UMAP
Full
Downsampled_reads
E
Marker Genes Selected
i
Marker Gene Intersection
5192
0
5815
5815
Downsampled_reads
11007
Full
Marker Genes
Markers Selected
ii
Marker Intersection
0
0
670047
670047
Full
670047
Downsampled_reads
Markers
logFC
iii
log2(Full Fold Change)
CCC = 0.40
log2(Downsampled_reads Fold Change)
PCA fit (excluding outliers): y=0.87x+-0.05
y=x
Adjusted p-value
iv
−log10(Full Adjusted p-value)
−log10(Downsampled_reads Adjusted p-value)

Supplemental Figure 8: Read downsampling to 4% of original size vs. full dataset in Scanpy, with a fraction that displays variation comparable to Seurat vs. Scanpy. (A) Filtering and HVG selection analysis UpSet plots consisting of overlap of sets of cells, genes, and HVGs. (B) PCA analysis through projection onto first 2 PCs, Scree plot eigenvalue comparison, and sine of eigenvectors. Black lines = point mapping between conditions. (C) KNN/SNN analysis through SNN neighborhood Jaccard index and degree ratio (full/-downsampled) per cell. (D) Clustering and UMAP analysis through UMAP plots of each condition, with alluvial plot showing cluster assignment mapping and degree of agreement. (E) Differential expression analysis through overlap of all significant ($p < 0.05$) marker genes across all clusters. (F) Differential expression analysis with aligned clusters of overlap of all markers. (G) Differential expression analysis with aligned clusters of logFC similarity. (H) Differential expression analysis with aligned clusters of adjusted p-value similarity.

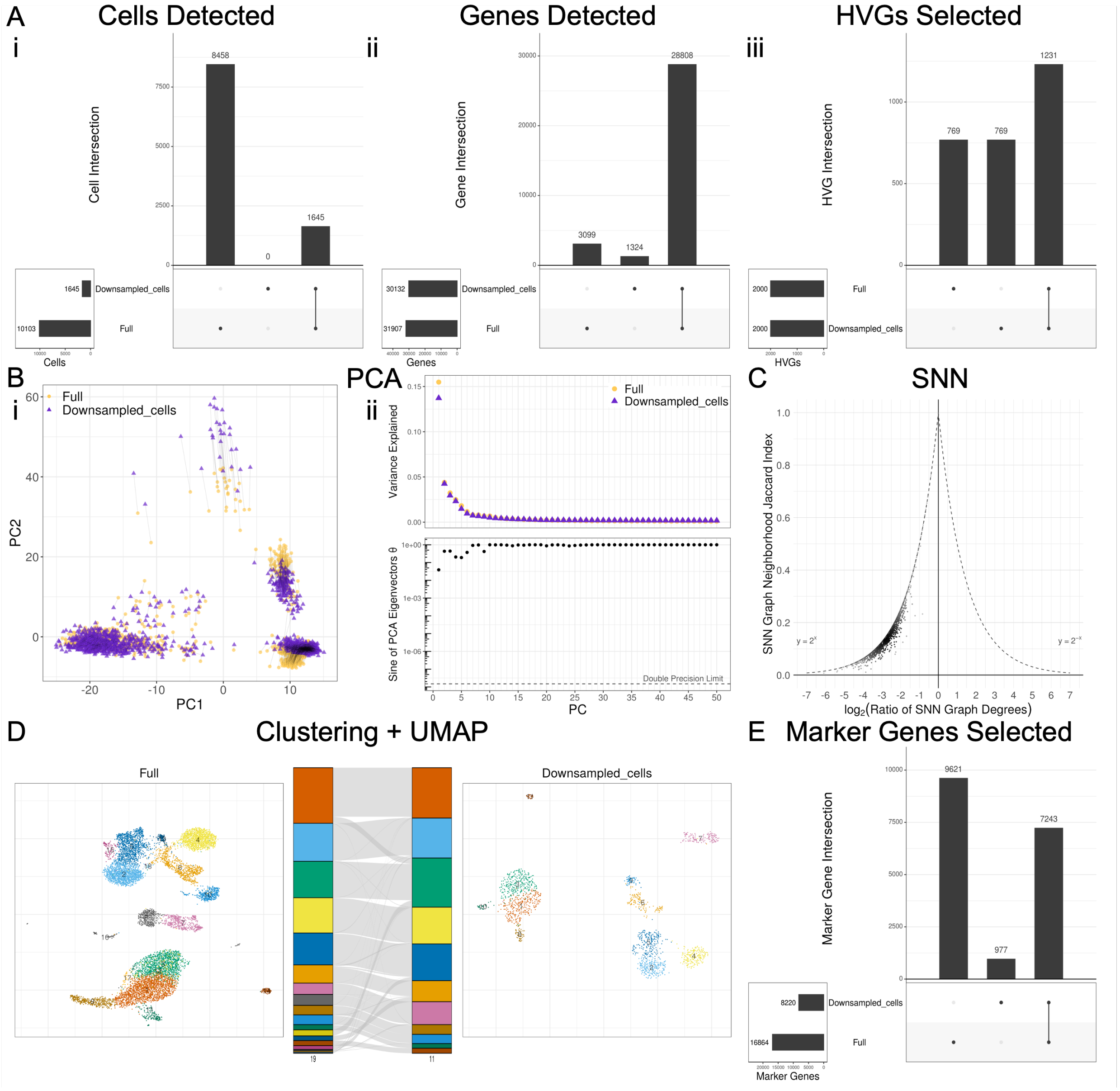

Supplemental Figure 9: Cell downsampling to 16% of original size vs. full dataset in Seurat, with a fraction that displays variation comparable to Seurat vs. Scanpy. (A) Filtering and HVG selection analysis UpSet plots consisting of overlap of sets of cells, genes, and HVGs. (B) PCA analysis through projection onto first 2 PCs, Scree plot eigenvalue comparison, and sine of eigenvectors. Black lines = point mapping between conditions. (C) KNN/SNN analysis through SNN neighborhood Jaccard index and degree ratio (full/downsampled) per cell. (D) Clustering and UMAP analysis through UMAP plots of each condition, with alluvial plot showing cluster assignment mapping and degree of agreement. (E) Differential expression analysis UpSet plot through overlap of all significant ($p < 0.05$) marker genes across all clusters.

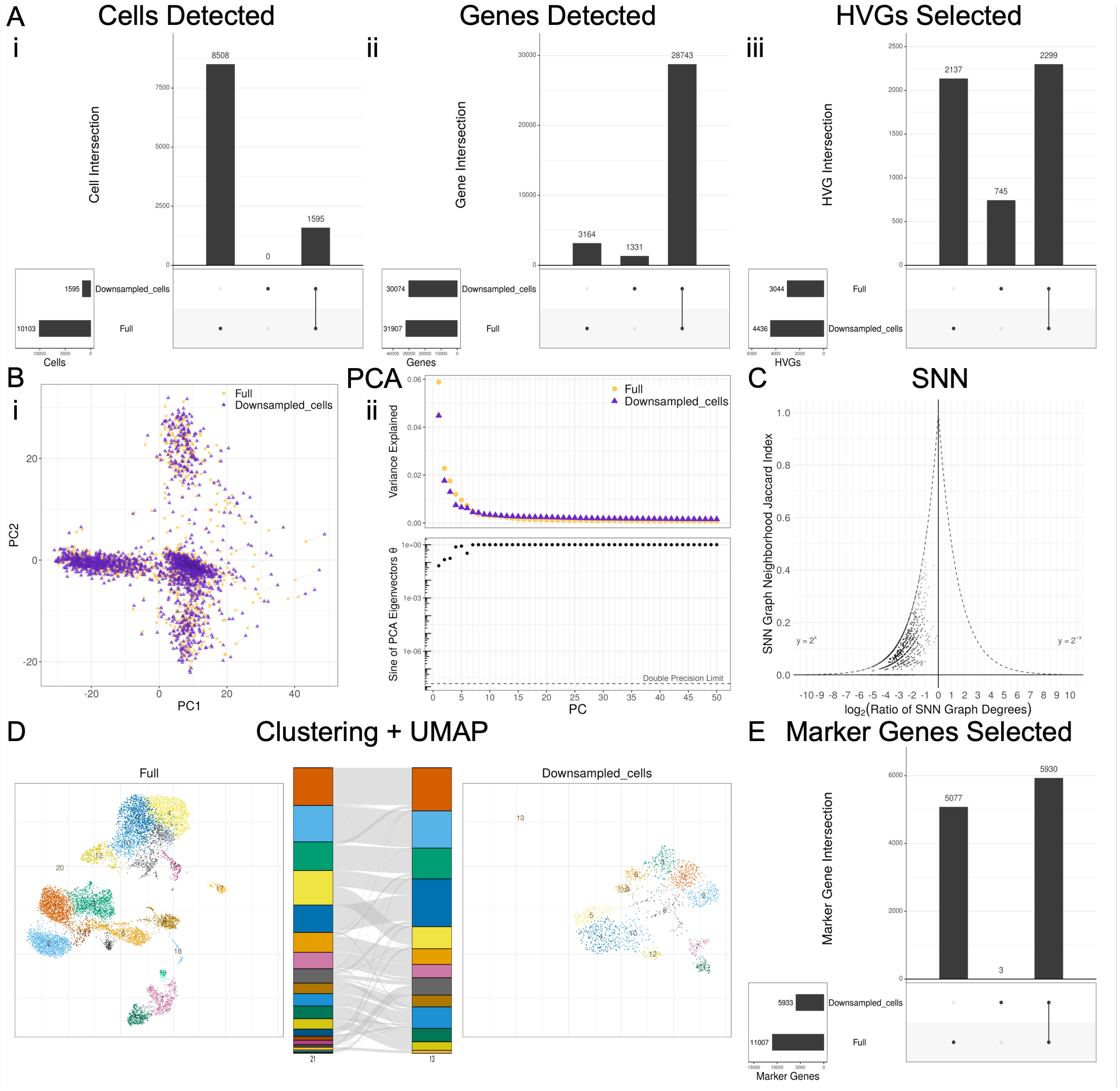

Supplemental Figure 10: Cell downsampling to 16% of original size vs. full dataset in Scanpy, with a fraction that displays variation comparable to Seurat vs. Scanpy. (A) Filtering and HVG selection analysis UpSet plots consisting of overlap of sets of cells, genes, and HVGs. (B) PCA analysis through projection onto first 2 PCs, Scree plot eigenvalue comparison, and sine of eigenvectors. Black lines = point mapping between conditions. (C) KNN/SNN analysis through SNN neighborhood Jaccard index and degree ratio (full/-downsampled) per cell. (D) Clustering and UMAP analysis through UMAP plots of each condition, with alluvial plot showing cluster assignment mapping and degree of agreement. (E) Differential expression analysis UpSet plot through overlap of all significant ($p < 0.05$) marker genes across all clusters.

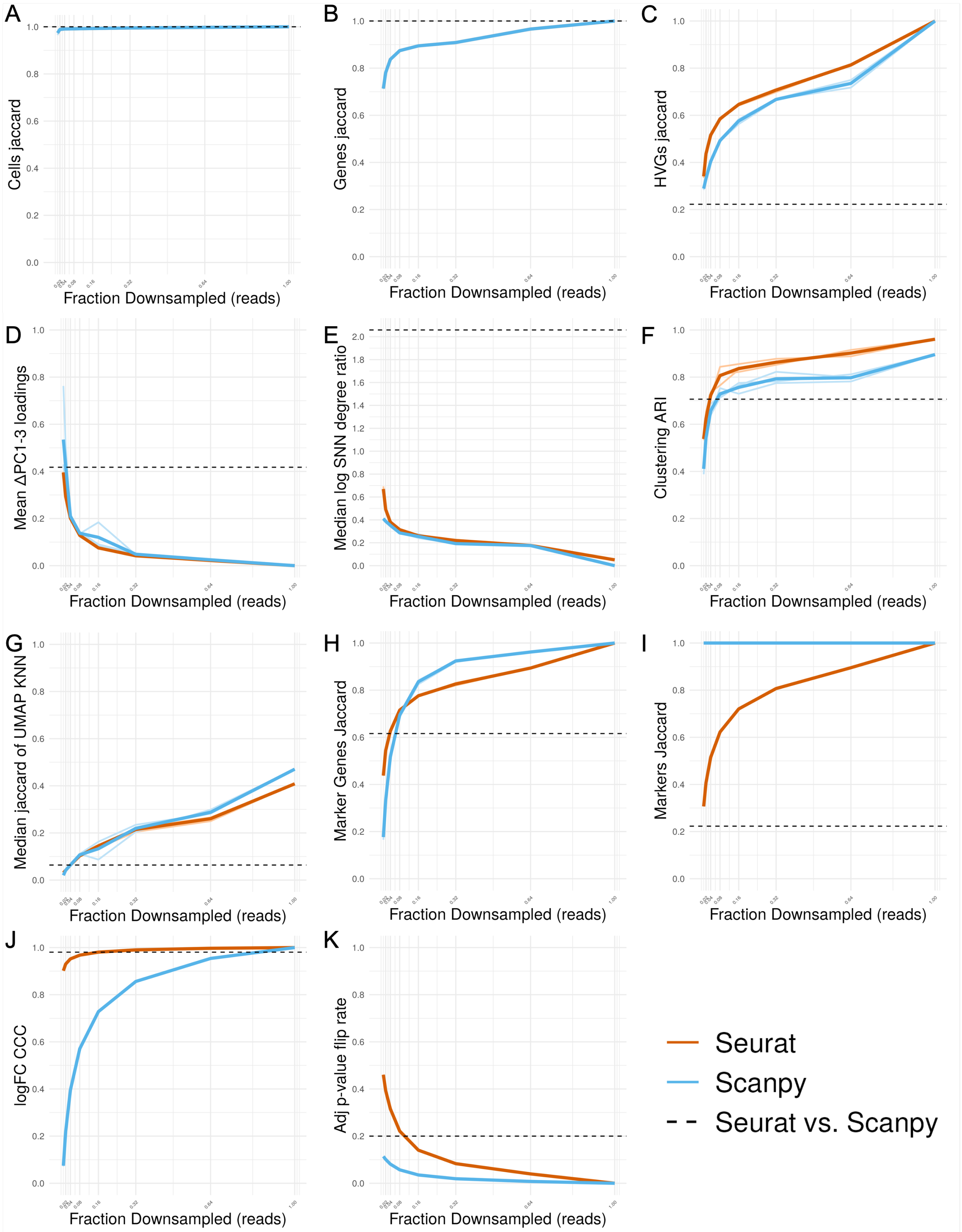
A
B
C
D
E
F
G
H
I
J
K
Cells jaccard
Genes jaccard
HVGs jaccard
Mean ΔPC1-3 loadings
Median log SNN degree ratio
Clustering ARI
Median jaccard of UMAP KNN
Marker Genes Jaccard
Markers Jaccard
logFC CCC
Adj p-value flip rate
Fraction Downsampled (reads)
Seurat
Scanpy
Seurat vs. Scanpy

Supplemental Figure 11: Metrics vs. fraction of reads downsampled. (A) Jaccard index of cell overlap. (B) Jaccard index of gene overlap. (C) Jaccard index of HVG overlap. (D) Mean of difference in corresponding PC loadings for PCs 1-3. (E) Median log(SNN degree ratio). (F) Adjusted Rand Index of clusters. (G) Median Jaccard index of UMAP-derived KNN graphs for each cell. (H) Jaccard index of sets of significant ($p < 0.05$) marker genes across all clusters. (I) Jaccard index of all markers. (J) CCC of logFC scatterploz. (K) Fraction of adjusted p-value that flipped across p=0.05 threshold. Orange = Seurat; Blue = Scanpy; lighter lines = replicates of downsampling across random seeds; dashed line = value recorded by Seurat vs. Scanpy with default settings.

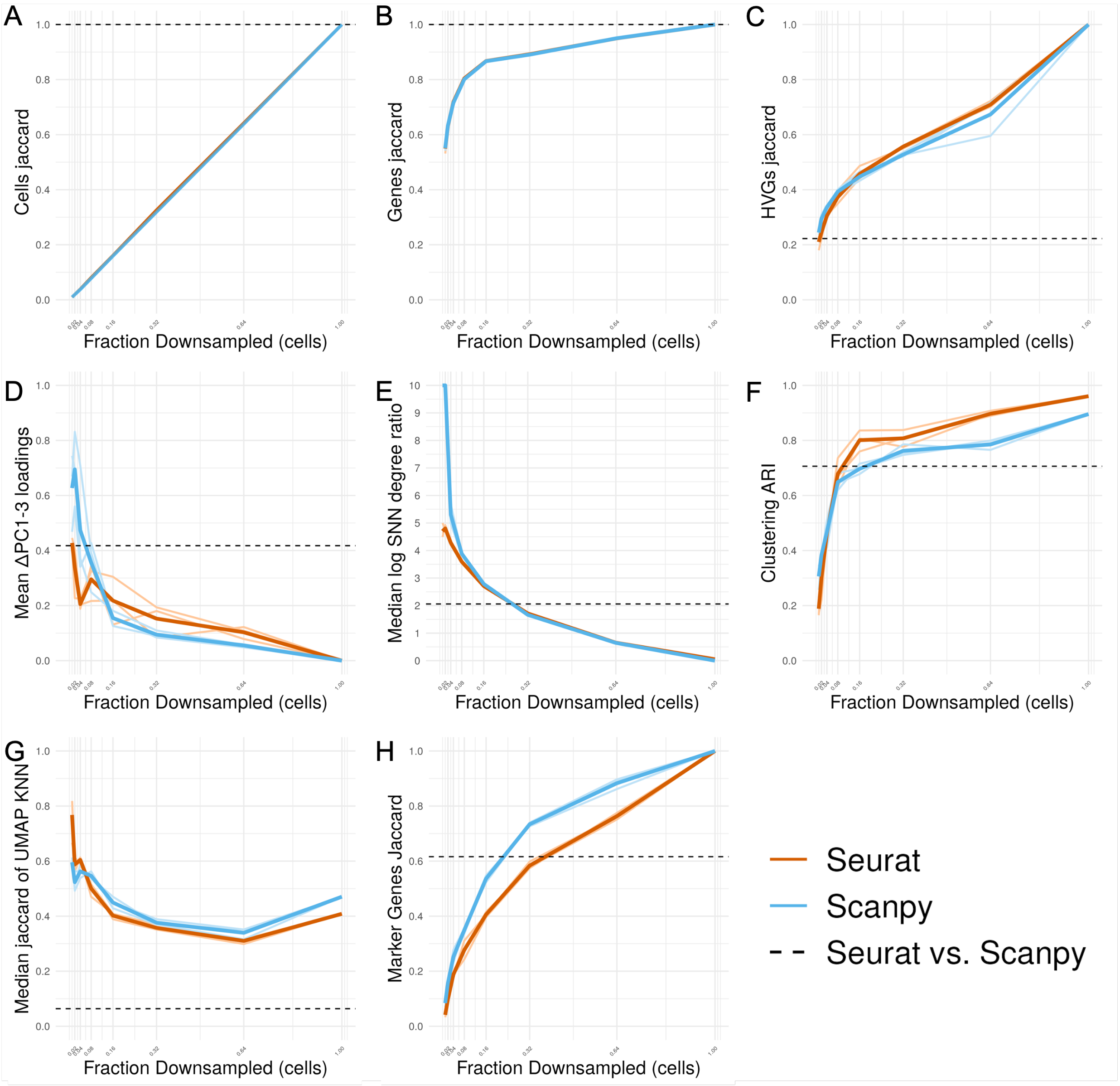

Supplemental Figure 12: Metrics vs. fraction of cells downsampled. (A) Jaccard index of cell overlap. (B) Jaccard index of gene overlap. (C) Jaccard index of HVG overlap. (D) Mean of difference in corresponding PC loadings for PCs 1-3. (E) Median log(SNN degree ratio). (F) Adjusted Rand Index of clusters. (G) Median Jaccard index of UMAP-derived KNN graphs for each cell. (H) Jaccard index of sets of significant ($p < 0.05$) marker genes across all clusters. (I) Jaccard index of all markers. (J) CCC of logFC scatterplot. (K) Fraction of adjusted p-value that flipped across p=0.05 threshold. Orange = Seurat; Blue = Scanpy; lighter lines = replicates of downsampling across random seeds; dashed line = value recorded by Seurat vs. Scanpy with default settings.

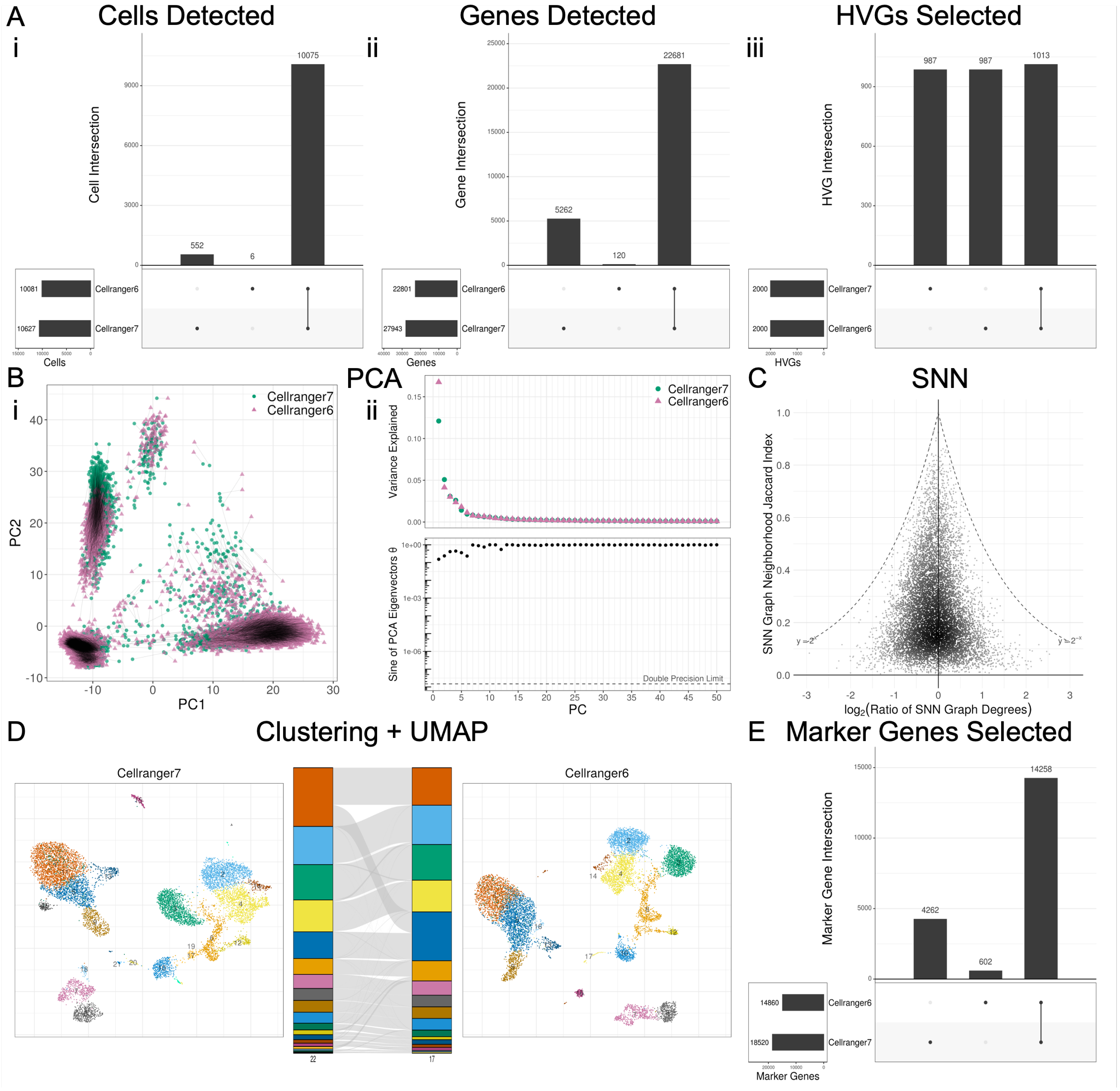

Supplemental Figure 13: Cell Ranger version control (v7 vs. v6). (A) Filtering and HVG selection analysis UpSet plots consisting of overlap of sets of cells, genes, and HVGs. (B) PCA analysis through projection onto first 2 PCs, Scree plot eigenvalue comparison, and sine of eigenvectors. (C) KNN/SNN analysis through SNN neighborhood Jaccard index and degree ratio (v7/v6) per cell. (D) Clustering and UMAP analysis through UMAP plots of each condition, with alluvial plot showing cluster assignment mapping and degree of agreement. (E) Differential expression analysis through overlap of all significant ($p < 0.05$) marker genes across all clusters.

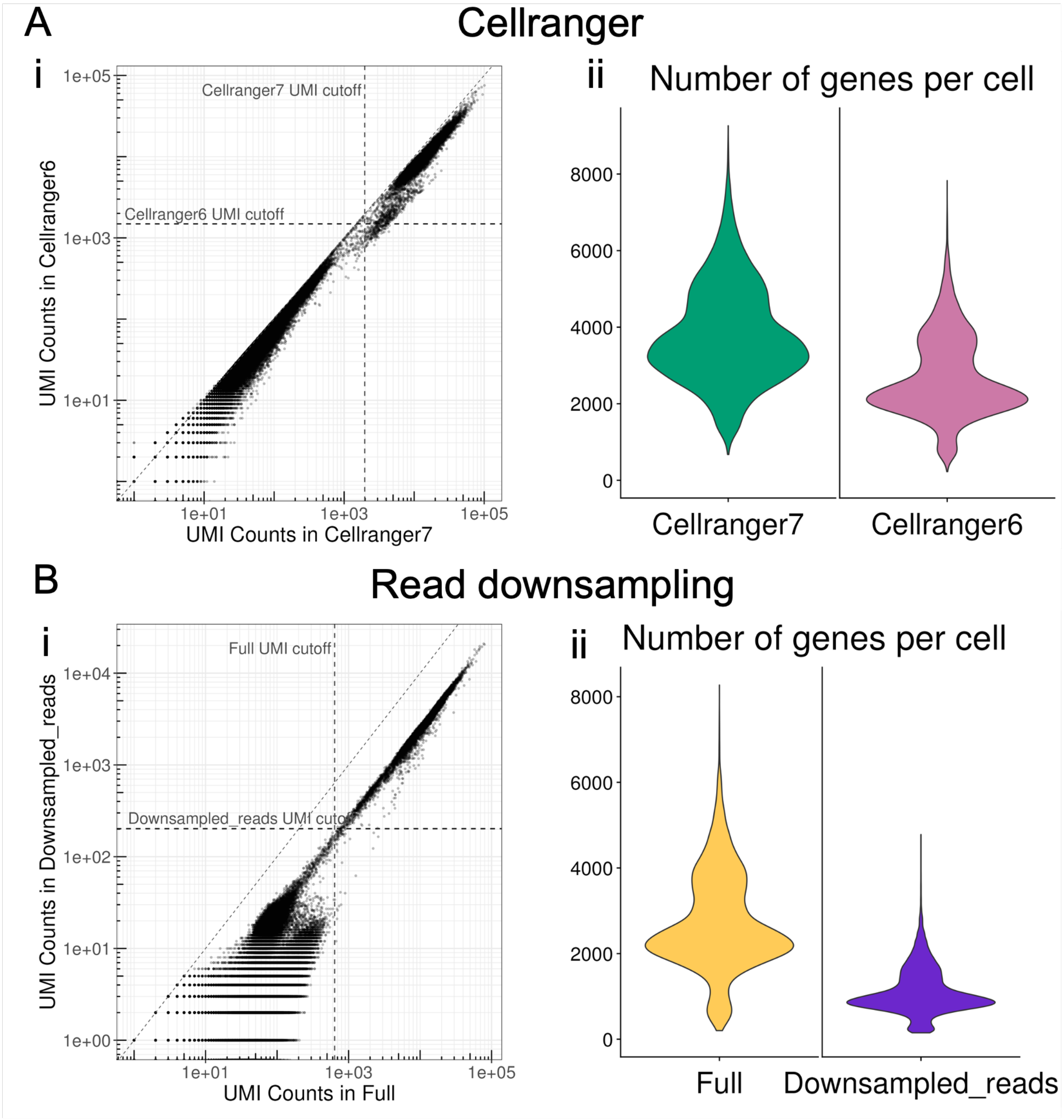

Supplemental Figure 14: UMI counts pre-filtering and number of features under different conditions. (A) Cell Ranger v7 vs. v6. (B) Full size dataset vs. downsampled reads to 4%.

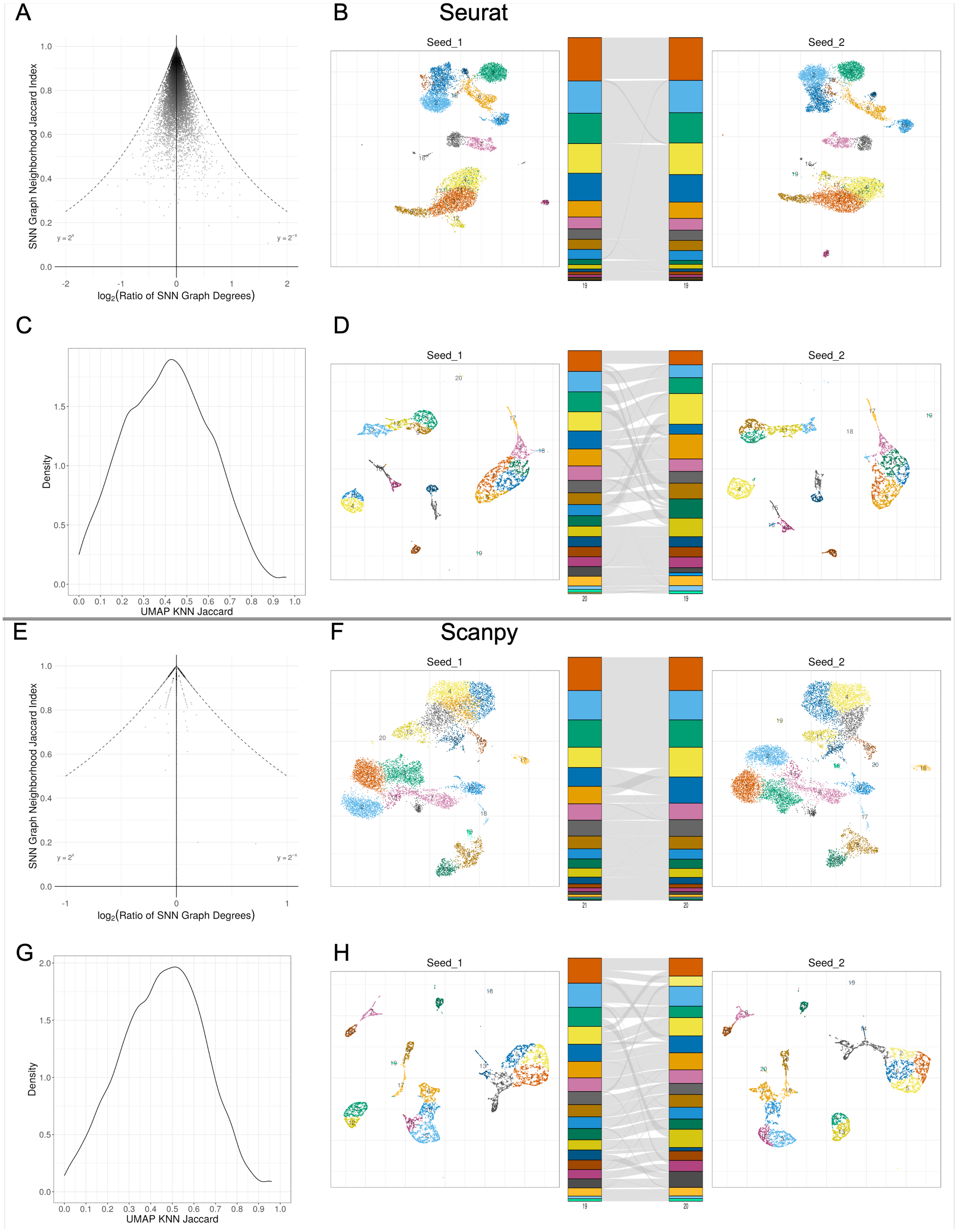

A
B
Seurat
Seed_1
Seed_2
SNN Graph Neighborhood Jaccard Index
log2(Ratio of SNN Graph Degrees)
y = 2^x
y = 2^-x
C
D
Density
UMAP KNN Jaccard
E
F
Scanpy
G
H

Supplemental Figure 15: Variability introduced by differences in random seeds for functions involving randomness. (A) Seurat SNN graph generation (Annoy), with different seeds for KNN graph generation. (B) Seurat clustering (Louvain), with different seeds for Louvain; and UMAP-projection (uwot), with different seeds for UMAP projection. (C) Seurat KNN graph generation from UMAP space, with different seeds for UMAP projection. (D) Seurat clustering (Leiden) and UMAP projection from UMAP space, with different seeds for UMAP projection. (E) Scanpy SNN graph generation (NNDescent), with different seeds for KNN graph generation. (F) Scanpy clustering (Leiden), with different seeds for Leiden; and UMAP-projection (UMAP-learn), with different seeds for UMAP projection. (G) Scanpy KNN graph generation from UMAP space, with different seeds for UMAP projection. (H) Scanpy clustering (Leiden) and UMAP projection from UMAP space, with different seeds for UMAP projection.

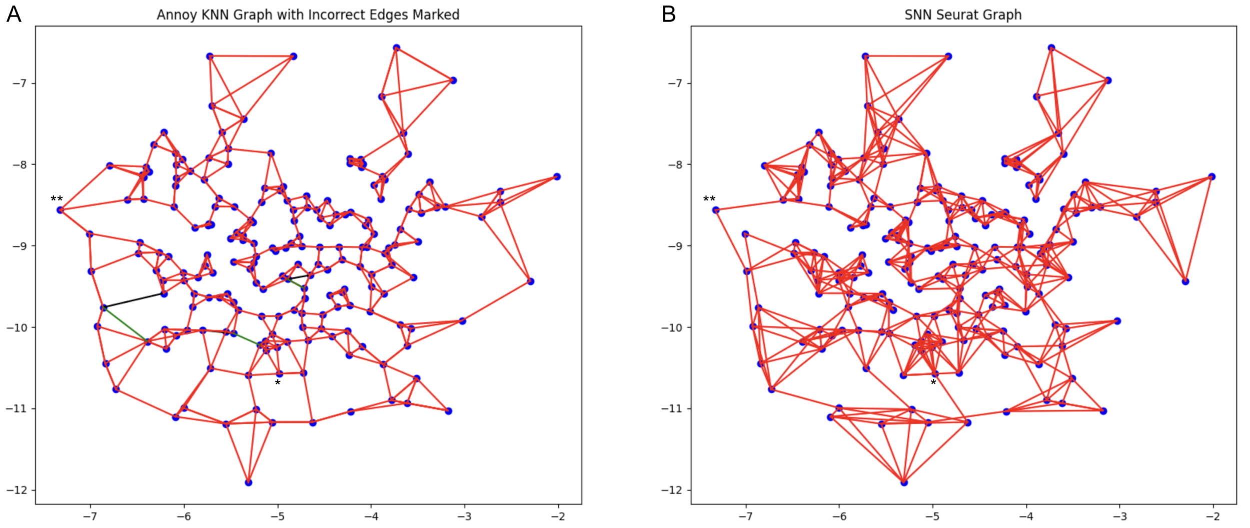

Supplemental Figure 16: Comparison of KNN and SNN graphs. (A) KNN graph (k=3) constructed with the approximate Annoy algorithm (directedness not shown). Black = incorrectly added edges; Green = incorrectly omitted edges. (B) SNN graph with Seurat's pruning method. An example hub node is marked with *. An example peripheral node is marked with **. Note: the SNN graph with Scanpy's method is simply the undirected KNN graph.

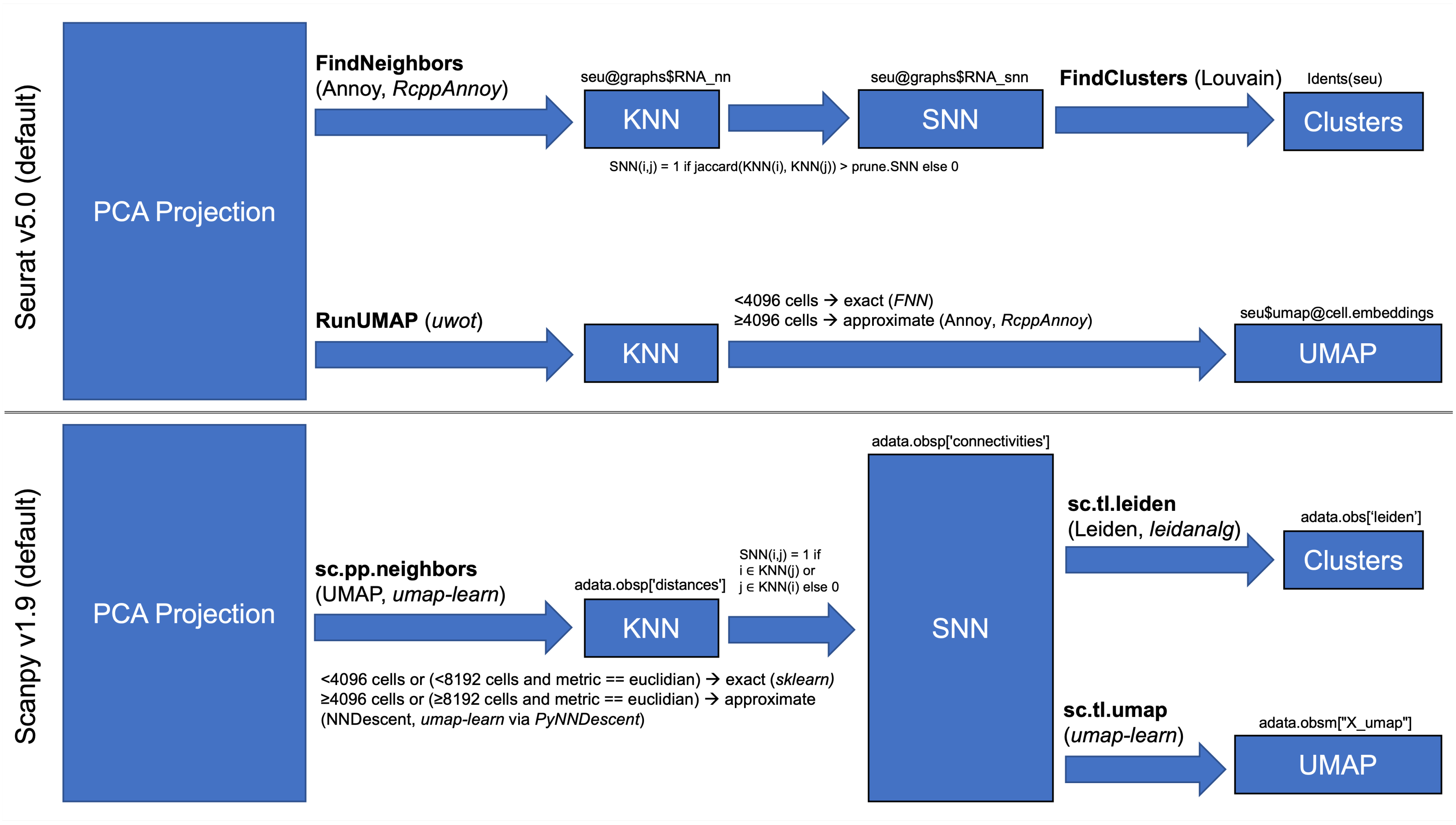
Seurat v5.0 (default)
PCA Projection
FindNeighbors
(Annoy, RcppAnnoy)
seu@graphs$RNA_nn
KNN
seu@graphs$RNA_snn
SNN
SNN(i,j) = 1 if jaccard(KNN(i), KNN(j)) > prune.SNN else 0
FindClusters (Louvain)
Idents(seu)
Clusters
RunUMAP (uwot)
KNN
<4096 cells → exact (FNN)
≥4096 cells → approximate (Annoy, RcppAnnoy)
seu$umap@cell.embeddings
UMAP
Scanpy v1.9 (default)
PCA Projection
sc.pp.neighbors
(UMAP, umap-learn)
adata.obsp['distances']
KNN
SNN(i,j) = 1 if
i ∈ KNN(j) or
j ∈ KNN(i) else 0
<4096 cells or (<8192 cells and metric == euclidian) → exact (sklearn)
≥4096 cells or (≥8192 cells and metric == euclidian) → approximate
(NNDescent, umap-learn via PyNNDescent)
adata.obsp['connectivities']
SNN
sc.tl.leiden
(Leiden, leidanalg)
adata.obs['leiden']
Clusters
sc.tl.umap
(umap-learn)
adata.obsm["X_umap"]
UMAP

Supplemental Figure 17: Schematic of protocol describing generation and downstream use of KNN and SNN graphs. Top is for Seurat, bottom is for Scanpy. KNN(i) refers to the full set of k-nearest neighbors for cell i. Bold = function/method name; Italics = outside package utilized.

**Seurat v5.0.2**	**Filtering**	**Normalization**	**HVG selection**	**Regression, Scaling**	**PCA**	**SNN**	**Clustering**	**UMAP**	**DE**
**Function nam**	CreateSeuratObject	NormalizeData	FindVariableFeatures	ScaleData	RunPCA	FindNeighbors	FindClusters	RunUMAP	FindAllMarkers
**Defaults**	**min.cells = 0**<br>**min.features = 0**	*scale.factor = 10000*<br>*normalization.method = "LogNormalize"*	selection.method = "vst"<br>**loess.span = 0.3**<br>*clip.max = "auto"*<br>*mean.function = FastExpMean*<br>*dispersion.function = FastLogVMR*<br>**num.bin = 20**<br>*binning.method = "equal_width"*<br>nfeatures = 2000<br>mean.cutoff = c(0.1, 8)<br>dispersion.cutoff = c(1, Inf)	**vars.to.regress = NULL**<br>*latent.data = NULL*<br>*split.by = NULL*<br>*model.use = "linear"*<br>*use.umi = FALSE*<br>*do.scale = TRUE*<br>**do.center = TRUE**<br>scale.max = 10	**npcs = 50**<br>*rev.pca = FALSE*<br>*weight.by.var = TRUE*<br>*approx = TRUE*<br>**features = NULL (HVGs)**	*reduction = "pca"*<br>dims = 1:10<br>k.param = 20<br>*prune.SNN = 1/15*<br>*nn.method = "annoy"*<br>*n.trees = 50*<br>**annoy.metric = "euclidean"**<br>nn.eps = 0<br>*l2.norm = FALSE*	*modularity.fxn = 1*<br>*initial.membership = NULL*<br>*node.sizes = NULL*<br>resolution = 0.8<br>method = "matrix"<br>algorithm = 1 (original Louvain)<br>*n.start = 10*<br>n.iter = 10<br>*group.singletons = TRUE*	umap.method = "uwot"<br>*n.neighbors = 30L*<br>**n.components = 2L**<br>*metric = "cosine"*<br>**n.epochs = NULL**<br>**learning.rate = 1**<br>min.dist = 0.3<br>**spread = 1**<br>*set.op.mix.ratio = 1*<br>*local.connectivity = 1L*<br>**repulsion.strength = 1**<br>**negative.sample.rate = 5**<br>a = NULL<br>b = NULL<br>dens.lambda = 2<br>dens.frac = 0.3<br>dens.var.shift = 0.1	*logfc.threshold = 0.1*[a]<br>test.use = "wilcox"<br>*min.pct = 0.01*[b]<br>*min.diff.pct = -Inf*<br>*only.pos = FALSE*<br>*max.cells.per.ident = Inf*<br>*latent.vars = NULL*<br>*min.cells.feature = 3*<br>*min.cells.group = 3*<br>*mean.fxn = NULL*<br>*return.thresh = 0.01*<br>*base = 2*
**(non-default) Arguments to match Scanpy defaults**			selection.method = "mean.var.plot"<br>mean.cutoff = c(0.0125, 3)<br>dispersion.cutoff = c(0.5, Inf)	vars.to.regress = c("nCount_RNA", "pct_mt")<br>scale.max = Inf		k.param = 15<br>dims = *total_pcs_used*	algorithm = "leiden"<br>resolution = 1.0	umap.method = "umap-learn"<br>min.dist = 0.5	logfc.threshold= 0<br>min.pct = 0<br>return.thresh = 1.0<br>min.cells.group = 1

[a]default changed from Seurat v4 (was logfc.threshold = 0.25)
[b]default changed from Seurat v4 (was min.pct = 0.1)

Supplemental Table 1: Seurat function details for scRNA-seq pipeline agreement with Scanpy. Bold = parameter in agreement by default; italics = no analogous paremeter in Scanpy; green = equivalent by default; yellow = equivalent with matched arguments; orange = partially incompatible, partially equivalent with matched arguments (depending on algorithm choice); red = incompatible

**Scanpy v1.9.5**	**Filtering**	**Normalization**	**HVG selection**	**Regression, Scaling**	**PCA**	**SNN**	**Clustering**	**UMAP**	**DE**
**Function name**	sc.pp.filter_cells, sc.pp.filter_genes	sc.pp.normalize_total	sc.pp.highly_variable_genes	sc.pp.regress_out, sc.pp.scale	sc.tl.pca	sc.pp.neighbors	sc.tl.leiden, sc.tl.louvain	sc.tl.umap	sc.tl.rank_genes_groups
**Defaults**	**min_cells = None**<br>**min_genes = None**<br>*max_cells = None*<br>*max_genes = None*<br>*min_counts = None (genes)*<br>*min_counts = None (cells)*<br>*max_counts = None (genes)*<br>*max_counts = None (cells)*	*target_sum = None*<br>*exclude_highly_expressed = False*<br>*max_fraction = 0.05*	n_top_genes = None<br>min_mean = 0.0125<br>max_mean = 3<br>min_disp = 0.5<br>max_disp = inf<br>**span = 0.3**<br>**n_bins = 20**<br>flavor = "seurat"	**zero_center = True**<br>max_value = None	**n_comps = 50** or 1 – minimum dimension size<br>zero_center = True<br>*svd_solver = "arpack"*<br>**use_highly_variable = None (HVGs)**	n_neighbors = 15<br>n_pcs = None<br>*use_rep = None*<br>*knn = True*<br>*method = "umap"*<br>**metric = "euclidian"**	resolution = 1<br>*restrict_to = None*<br>*directed = True*<br>*use_weights = True*<br>n_iterations = -1<br>*partition_type = None*<br>*flavor = "vtraag" (louvain)*	min_dist = 0.5<br>**spread = 1.0**<br>*n_components = 2*<br>**maxiter = None**<br>**alpha = 1.0**<br>**gamma = 1**<br>**negative_sample_rate = 5**<br>*init_pos = "spectral"*<br>**a = None**<br>**b = None**<br>method = "umap"	*use_raw = None*<br>*reference = "rest"*<br>n_genes = None<br>rankby_abs = False<br>pts = False<br>method = None<br>corr_method = 'benjamini-hochberg'<br>*tie_correct = False*
**(non-default) Arguments to match Seurat defaults**			n_top_genes = 2000<br>flavor = "seurat_v3"	Omit sc.pp.regress_out<br>max_value = 10	zero_center = False	n_neighbors = 20<br>n_pcs = 10<br>use_rep = 'X_pca'	function sc.tl.louvain instead of sc.tl.leiden<br>resolution = 0.8<br>n_iterations = 10	min_dist = 0.3	use_raw=True<br>pts = True<br>method = "wilcoxon"<br>corr_method = "bonferroni"<br>tie_correct = True<br>manual filtering (abs(log_fc) ≥ 0.1, pts ≥ 0.01, pts_rest ≥ 0.01 p_value < 0.01)

Supplemental Table 2: Scanpy function details for scRNA-seq pipeline agreement with Seurat. Bold = parameter in agreement by default; italics = no analogous paremeter in Seurat; green = equivalent by default; yellow = equivalent with matched arguments; orange = partially incompatible, partially equivalent with matched arguments (depending on algorithm choice); red = incompatible
